# Promoting motivation and reducing stress in medical students by utilizing self-determination theory – a randomized controlled trial in practical psychiatry courses

**DOI:** 10.1186/s12909-024-06181-9

**Published:** 2024-10-19

**Authors:** Nina Triebner, Franziska Sonnauer, Miriam Rauch, Gian-Marco Kersten, Christoph Rauch, Stefan Mestermann, Maximillian Bailer, Johannes Kornhuber, Janine Utz, Philipp Spitzer

**Affiliations:** 1 Department of Psychiatry and Psychotherapy, Friedrich-Alexander-Universität Erlangen-Nürnberg, Schwabachanlage 6, 91054 Erlangen, Germany; 2 Department for Vascular Surgery, Neumarkt in der Oberpfalz, Neumarkt Hospital, Neumarkt in der Oberpfalz, Germany; 3 Clinic for Psychiatry, Engelthal Hospital, Engelthal, Germany

**Keywords:** Intrinsic motivation, Extrinsic motivation, Self-determination theory (SDT), Stress, Medical education, Psychiatry, Randomized controlled trial (RCT)

## Abstract

**Background:**

Medical students experience high levels of stress and related mental health problems. Students’ autonomous and controlled motivation and their mental well-being are interconnected. This study aimed to investigate whether an innovative teaching concept based on self-determination theory (SDT) could improve students’ motivation and thereby reduce their stress levels, ultimately providing a healthier framework for learning.

**Methods:**

In a week-long practical psychiatry course for medical students, a new didactic concept was implemented in half the groups (*n* = 73) and compared with the preexisting concept (*n* = 75) as a randomized controlled trial (RCT). To promote the SDT-target factors of perceived autonomy, competence, and relatedness, the methods used included team building, exclusively positive feedback, group discussions, and choice in task distribution. Significant group differences in motivation, stress, performance, and their relationships were analyzed through *t*-tests, multiple linear regression analyses, mediation analyses, and hierarchical linear modeling (HLM) using questionnaires collected before (t_0_) and after (t_1_) the course, and students’ exam results (t_2_).

**Results:**

In the innovation group (*n* = 53), intrinsic motivation/interest (*d* = 0.41; *p* = .019) and perceived choice/autonomy (*d* = 0.33; *p* = .048) were greater than in the control group (*n* = 52). While autonomous regulation remained stable, the innovation group showed reduced controlled regulation (*d* = -0.36; *p* = .033) and reported significantly lower stress (*d* = -0.55; *p* = .003). The observed changes in motivation collectively mediated the stress reduction. However, students in the innovation group achieved lower exam scores, which seemed to result from the absence of critical feedback, but not from the observed differences in motivation or stress.

**Conclusions:**

This study demonstrated that enhancing intrinsic motivation through SDT-based teaching can effectively reduce stress in medical students. Exclusively strengths-based positive feedback may have hindered exam performance, but optimizing educational concepts to promote motivation and reduce stress will be a valuable step toward improving medical students’ mental well-being.

**Supplementary Information:**

The online version contains supplementary material available at 10.1186/s12909-024-06181-9.

## Background

The demanding environment of medical schools and the high levels of distress experienced by medical students are topics frequently discussed both in specialist circles as well as the public debate. High rates of depression, burnout, and anxiety among medical students have been reported in various studies worldwide over the past decades [1, 2]. Considering students’ mental well-being and its effects on patient care, it is therefore imperative that educators and institutions find effective solutions to provide students with a learning environment that promotes mental well-being and reduces stress [3, 4].

Intrinsic motivation is known to be a decisive factor in promoting students’ mental and physical well-being [5–9]. The self-determination theory (SDT) encompasses a basic needs theory, which asserts that intrinsic motivation is sustained by satisfying three fundamental psychological needs: autonomy, competence, and relatedness [10–14]. Autonomy refers to the need for individuals to experience self-direction and personal agency in their actions and decisions. When learners feel they can make meaningful choices, they are more likely to engage in learning activities out of genuine interest [10–14]. Competence is the feeling of being effective and capable in one’s endeavors. In an academic setting, providing students with tasks that are challenging but achievable, along with positive reinforcement, fosters their sense of proficiency and mastery [10–14]. Finally, relatedness emphasizes the importance of interpersonal connections. When students feel understood, valued, and supported by their peers and instructors, they are more likely to be motivated and engaged [10–14]. The integration of these three needs in a teaching environment, which encourages students’ interests, provides supportive feedback, choices, and room for collaboration, as well as reflection, can foster intrinsic motivation [15–19] and reduce stress in an educational setting [20, 21]. Furthermore, it has been demonstrated that a lack of intrinsic motivation is associated with depression and stress in undergraduate students [22]. Moreover, intrinsic motivation leads to the use of more deep learning [23–25] as well as better academic performance [5, 9, 11, 25–28].

The aim of our study was to investigate whether intrinsic motivation could be promoted in medical education by implementing a set of didactic methods designed to support students’ basic psychological needs in accordance with SDT. These included moderated group discussions, team building exercises, giving students freedom of choice regarding their individual focal topics, and implementing exclusively strengths-based positive feedback. We also examined whether intrinsic motivation would in turn contribute to a reduction in medical students’ experience of stress during a one-week psychiatric practical course. Additionally, we analyzed whether the new course concept would enable students to achieve exam results equal to their peers in the control group despite dedicating a part of the course time to these methods and not giving specific critical feedback.

Hypotheses:

1. An innovative teaching method optimized according to the SDT can promote students’ intrinsic motivation.
2. Furthermore, it can facilitate lower stress levels.
3. A favorable development of motivational factors can lead to a reduction in stress.
4. The level of performance in exams remains unchanged.

## Methods

### Participants, course structure and ethical approval

Fourth-year medical students completing a mandatory five-day practical course at our psychiatric clinic participated in this study in the summer semester of 2022.

The one-week course was offered during most weeks throughout the semester and in the early part of the semester break. Students were free to sign up for their preferred time slot online on a first-come, first-serve basis at the beginning of the semester. The groups of five to eight students were subsequently randomized so that half of them were taught according to the usual didactic concept and the other half using an innovative concept improved according to the principles of the SDT. Lecturers were informed which study arm they were teaching. To ensure consistent implementation of the study protocol, they received a briefing on the procedures relevant to their group, along with a handbook containing precise instructions, dos and don’ts, and a comprehensive timetable.

The students were blinded in the sense that they were not informed which study arm they were participating in. All students took part in the survey voluntarily and were explicitly informed that participation would not impact their exam grades and that the data would be processed anonymously.

This study was submitted to the Institutional Review Board (ethical committee) of the Friedrich-Alexander University Erlangen-Nuremberg and received a designation of exempt according to § 15 BO (professional code of conduct for the physicians of Bavaria). Therefore, a need for consent to participate was deemed unnecessary by the afore mentioned university’s review board according to national regulations.

### Course concept

The course comprised five consecutive afternoons of practical teaching (Monday to Friday, 4–5 h daily, overall 21 h) at our psychiatric clinic and concluded with an objective structured clinical examination (OSCE) the following Monday. Prior to on-site practical education, students completed a multimedia online course (10 h) at their own pace, providing a theoretical background for the practical part.

Every day during on-site teaching, real and simulated patients were each interviewed by one of the students. Thereafter, the simulated patients, fellow students, and the lecturer provided feedback on the student’s performance. During the OSCE, students were given a standardized patient scenario and graded on taking the medical history, applying communication models, and giving a case report.

Our innovation encompassed four newly implemented methods (Table 1).

1. At the beginning and end of each course day, students gathered with their lecturer for a five-minute structured group discussion with an extended 30-minute introductory round on their first day and a 15-minute concluding discussion at the end of the last day. The aim was to provide an open, supportive environment where students felt comfortable expressing their emotions and concerns. Additionally, they were given a space to reflect on expectations for the course, their learning process, and takeaways from each day, as well as positive and potentially difficult experiences throughout the course and with psychiatric patients encountered in their education and career thus far. The method was implemented to support the basic need for relatedness by promoting group cohesion and psychological safety, thereby enhancing students’ sense of belonging. This process also contributed to autonomy support by encouraging students to experience task relevance and understand the rationale of their learning activities in their personal and professional development. In contrast, the control group did not engage in structured group discussions, reducing opportunities for students to connect with each other and the lecturer on a personal level and to reflect on task relevance and rationale.
2. Instead of having a scheduled break to use as they wish, all students engaged in a group activity to further support the need for relatedness by promoting cooperation and train their communication skills. All activities were designed to create a space for teamwork and reflection on collaborative learning. Students were, for instance, asked to coordinate through non-verbal communication when counting up to 30 or to work together in lowering a long stick to the floor while supporting it on both their pointer fingers at all times. The control group, by contrast, did not engage in these structured activities, which may have limited the development of interpersonal connections.
3. To strengthen their sense of autonomy, our innovation group students were able to freely distribute the topics, patients, time slots, and number of interviews among themselves. This allowed students to have a sense of choice and ownership over their learning experience, which is a key factor in fostering intrinsic motivation. On the other hand, the control group had their interviews assigned by the lecturer in advance, which restricted their autonomy and may have reduced their sense of control over the learning process.
4. Lastly, students, teachers, and simulated patients in the innovation group were instructed to give only strengths-based positive feedback. Receiving positive feedback has been shown to enhance students’ perceived competence [8] as well as motivation and confidence [29]. By pointing out which aspects were particularly well handled by the interviewer, without giving critical or negative feedback on weaker aspects of their performance, we aimed to improve the student’s sense of competence. Additionally, the goal was to reduce pressure and stress caused by fear of negative responses to their performance, thereby also strengthening the group’s relatedness. The control group followed the guideline of giving critical constructive feedback to the interviewer, mentioning both positive and negative aspects of their performance. The method may fall short in fully supporting competence, as students may leave feeling less sure of their abilities after receiving detailed critiques of their performance and weaknesses by the group.

**Table 1:** Differences in the didactical concepts of the innovation group and the control group and the respective target factors of self-determination theory (SDT)

	Innovation	Control	Target factor
1) Framework	Moderated group discussions twice daily	No structured framework	relatedness, autonomy
2) Breaks	Team building tasks	Breaks at students’ free disposal	relatedness
3) Case distribution	Cases distributed by the students	Cases assigned by the lecturer	autonomy
4) Feedback	Strengths-based positive feedback	Critical constructive feedback	competence, relatedness

### OSCE testing

Simulated patients performed one of four standardized clinical cases (depression, dementia, schizophrenia, or obsessive-compulsive disorder). Students were graded by examiners that differed from their lecturers. To facilitate a fair exam, we used a standardized rubric, rating the student’s performance out of 60 points in conducting a mental status examination, including taking the psychopathological history, using communication models, and giving a conclusory case presentation. The point score is used for research purposes and to monitor the didactic quality of the course, while only a pass (≥ 36 points) or fail grade is registered on students’ grade record. The same OSCE had been conducted for several past semesters at our psychiatric university hospital, showing no improvement in the overall results over time, which indicates that previous knowledge of the exam structure is not advantageous to students’ performance; rather, their actual competence is examined [30].

### Questionnaires

**Fig. 1:**
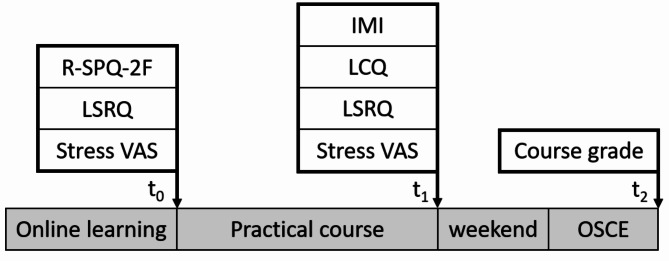
Data collected before (t0) and after (t1) the course week included the revised 2-factor study process questionnaire (R-SPQ-2 F), the learning self-regulation questionnaire (LSRQ), the task evaluation questionnaire of the intrinsic motivation inventory (IMI) and the perceived autonomy support learning climate questionnaire (LCQ) as well as self-reported stress on a visual analog scale (Stress VAS) and the course grade achieved in the objective structured clinical exam (OSCE) on Monday after the course week

The students answered previously published and well-established questionnaires online before starting their first day of practical teaching and again at the end of the last day of the course, three days before the OSCE (Fig. 1). All questionnaires were administered in German, the language of instruction, to prevent potential language-related inaccuracies.

- Before the course, we collected the revised 2-factor study process questionnaire (R-SPQ-2 F) with 20 items to measure students’ deep and surface learning approaches [31]. It comprises the subscales of deep and surface motive, as well as deep and surface strategy, which are evaluated via a 5-point Likert scale [1 (never/rarely true) to 5 (always/almost always true)] and added up to calculate each approach score [31]. The R-SPQ-2 F has been validated and shown to be reliable, with reported Cronbach’s alpha values of 0.73 for deep approach and 0.64 for the surface approach subscale [31]. The questionnaire was used to evaluate how students’ deep and surface learning approaches would influence students’ performance compared to factors directly targeted by our course concept.
- We collected the interviewing version of the learning self-regulation questionnaire (LSRQ) [32] both before and after the course. It comprises 14 items on the subscales “autonomous regulation”, reflecting intrinsic motivation, and “controlled regulation”, reflecting extrinsic motivation, based on a 7-point Likert scale [1 (not at all true) to 7 (very true)]. In past studies the alpha reliabilities for these two subscales have been approximately 0.75 for controlled regulation and 0.80 for autonomous regulation. Validation was done at the level of these two categories [32]. The questionnaire allows the calculation of a “relative autonomy index” by subtracting controlled from autonomous regulation subscale scores [32]. It was used examine differences in students’ styles of regulation in our two study arms after the course while being able to control for their initial values from before the course.
- Before and after the course, we assessed stress levels during the past week using a visual analog scale (VAS) operationalized from 0 to 100, so that we could compare the values after the course in the two study arms and account for their base line before the course. Construct validity of VAS for assessing stress has been established through comparison with other well-established tools for measuring stress [33, 34].
- At the end of the course, students answered the task evaluation questionnaire of the intrinsic motivation inventory (IMI), which comprises four subscales [35]. The “interest/enjoyment” subscale is considered a self-reported measure of intrinsic motivation. “Perceived competence”, “perceived choice”, which reflects a sense of autonomy, and “pressure/tension” are considered predictors of intrinsic motivation. Each of the 22 items is evaluated on a 7-point Likert scale [1 (not at all true) to 7 (very true)] [35]. There has been found strong support for the validity of the IMI as well as adequate reliability of its subscales [36]. The questionnaire was used to examine differences between the two study arms after the course, as well as interactions between the different factors and motivation.
- Additionally, we collected the perceived autonomy support learning climate questionnaire (LCQ) with 15 items after the course, which also uses a 7-point Likert scale [1 (not at all true) to 7 (very true)] [37]. The validated questionnaire has shown to be reliable, with reported Cronbach’s alpha values of 0.71 [38]. It was used to evaluate whether the differences in didactical concepts between the two groups could be detected in their perceived autonomy support by and sense of relatedness to the lecturer.
- Finally, we asked the students to give freeform feedback about their experience with the course with a separate question each for positive and negative remarks to clearly distinguish between their evaluation of the mentioned factors. Meanwhile, without limiting answers to certain topics or aspects of the course, students were free to report on exactly the points they felt were important to them.

### Data analysis

The data were analyzed through *t*-tests, multiple linear regressions, simple and parallel mediations, and hierarchic linear modeling.

Regression analyses and *t*-tests were performed using SPSS (SPSS^®^ 29, IBM^®^, Armonk, USA). Assumptions of linearity, normality, and homoscedasticity were assessed, ensuring the appropriateness of the models. We calculated effect sizes (*d*) for group differences [39] with their confidence intervals (*CI*) and applied the empirically derived guidelines of 0.15 being a small effect, 0.36 a medium effect, and 0.65 a large effect in social psychology [40]. The models’ goodness-of-fit was interpreted according to Cohen [39].

Mediation analyses were performed using the PROCESS macro by Hayes (PROCESS Procedure for SPSS^®^ Version 4.2, Andrew F. Hayes, Calgary, Canada), which uses ordinary least squares regression, yielding unstandardized path coefficients for total, direct, and indirect effects. Bootstrapping with 5000 samples together with heteroscedasticity-consistent standard errors [41] was employed to compute the confidence intervals and inferential statistics. The effects were deemed significant when the confidence interval for the indirect effect did not include zero. Visual inspection of scatterplots after LOESS smoothing indicated an approximately linear relationship between all variables involved.

We used hierarchic linear modeling or HLM (HLM^®^ 8, Scientific Software International, Inc., Skokie, USA) with level-1 variables describing data collected from a single student and level-2 variables comprising data collected in the same group (same lecturer, time slot, examiner, study arm). The results of the hierarchic linear modeling are reported as the difference between slopes (*b*) of the regression curves and its significance (*p*).

Statistical significance was set at *p* ≤ .05.

## Results

### Participant characteristics

In the summer semester of 2022, 148 medical students participated in the course, 73 of whom participated in a group with the new didactical concept and 75 of whom participated in a control group with our conventional concept. We obtained complete sets of data from 105 students (return rate: 71%), comprising the questionnaires taken before (t_0_) and after the course (t_1_) as well as the results from the OSCE (t_2_). Notably, for variables assessed at t_0_, no statistically significant group differences were found using two-sided *t*-tests (Table 2). Nonetheless, preexisting non-significant but potentially relevant group differences, such as stress scores, were controlled for in our subsequent data analysis.

**Table 2:** Characteristics of the study participants at t0 before the course; values are given as the mean (± standard deviation)

		Innovation	Control
*N* (female/male/other)		53 (41/11/1)	52 (37/15/0)
Age [years]		24.0 (± 3.1)	24.3 (± 2.9)
Study duration [semesters]		8.1 (± 0.3)	8.1 (± 0.2)
R-SPQ-2 F	Deep motive	3.40 (± 0.57)	3.36 (± 0.52)
	Deep strategy	3.13 (± 0.49)	3.19 (± 0.49)
	Surface motive	2.38 (± 0.57)	2.53 (± 0.59)
	Surface strategy	2.72 (± 0.63)	2.93 (± 0.61)
LSRQ	Autonomous regulation	6.02 (± 0.66)	5.86 (± 0.72)
	Controlled regulation	3.97 (± 1.00)	3.96 (± 0.91)
	Relative autonomy index	2.05 (± 1.23)	1.90 (± 1.16)
Stress		42.89 (± 25.09)	52.23 (± 23.55)

### Greater intrinsic motivation, higher perceived autonomy, and less controlled regulation in the innovation group

With respect to our hypothesis that a teaching concept according to the SDT could promote students’ intrinsic motivation, we conducted one-sided *t*-tests to examine group differences at the end of the course. Our control group served as the reference group.

**Fig. 2:**
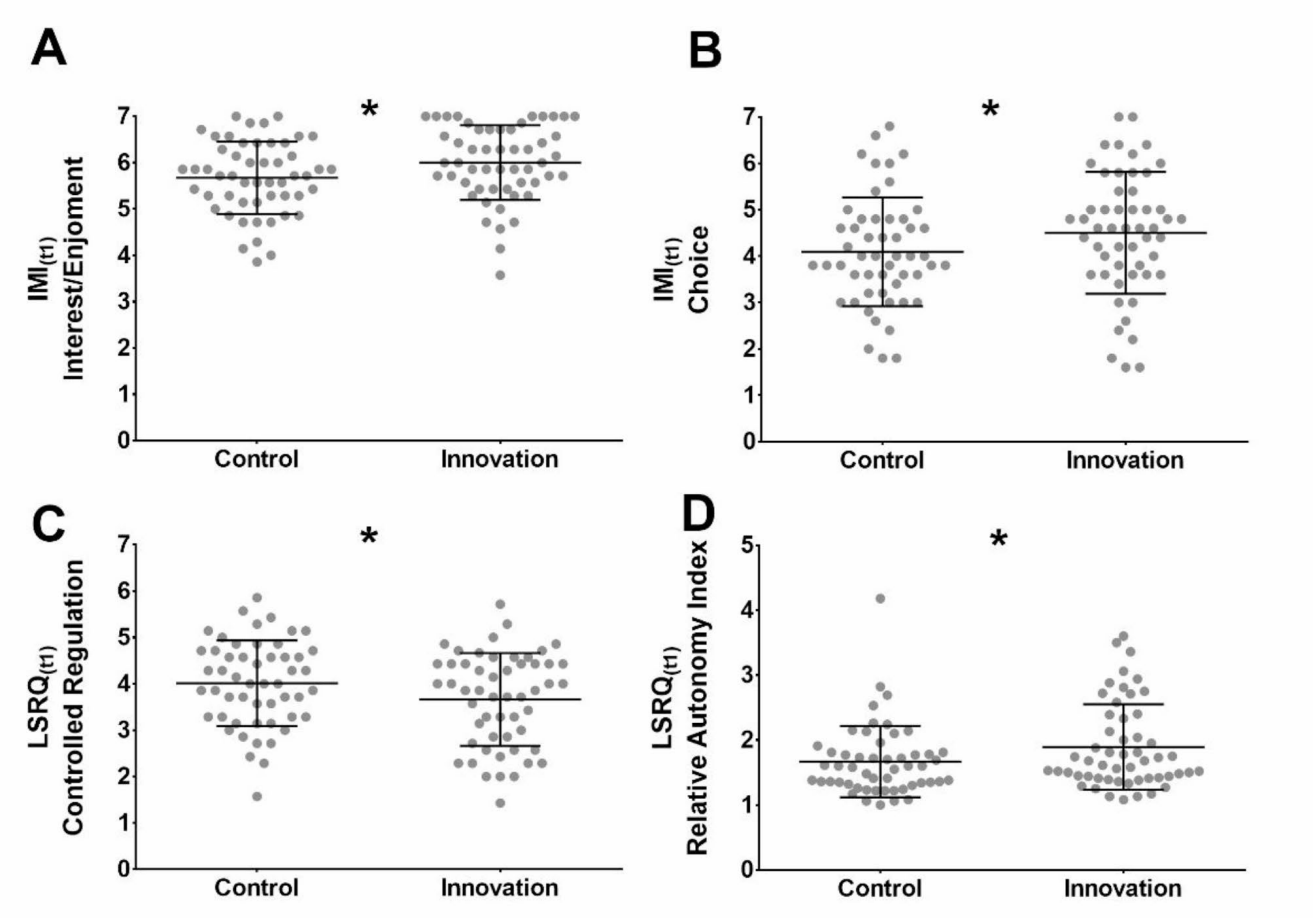
Significant differences between the control and innovation group were found after the course week (t1) regarding intrinsic motivation measured on the interest/enjoyment subscale of the intrinsic motivation inventory (IMI) (*p* = .019) and perceived choice (*p* = .048) measured on the IMI’s choice subscale. Further significant differences were found on the learning self-regulation questionnaire (LSRQ) subscale of controlled regulation (*p* = .033) and its relative autonomy index (*p* = .037), which is calculated by subtracting the controlled regulation subscore from the questionnaire’s autonomous regulation subscore. All (sub)scores shown are measured on a 7-point Likert scale

After the course (t_1_), students in the innovation group showed significantly greater levels of intrinsic motivation regarding the course, as measured by the IMI (*M*_C_ = 5.67 ± 0.78; *M*_I_ = 6.00 ± 0.81; *p* = .019), with a standard error difference of 0.16 (Fig. 2A). The effect size for this difference was medium (*d* = 0.41; 95% *CI* [0.02, 0.80]).

Similarly, a greater sense of choice (IMI) was observed in the innovation group at t_1_ (*M*_C_ = 4.09 ± 1.17; *M*_I_ = 4.50 ± 1.32; *p* = .048), with a standard error difference of 0.24 (Fig. 2B). The effect size was small (*d* = 0.33; 95% *CI* [-0.06, 0.71]).

Additionally, the innovation group scored significantly lower on the LSRQ’s controlled regulation subscale at t_1_ (*M*_C_ = 4.01 ± 0.92; *M*_I_ = 3.66 ± 1.00; *p* = .033), with a standard error difference of 0.19 (Fig. 2C). The effect size for this difference was medium (*d* = -0.36; 95% *CI* [-0.75, 0.02]).

Correspondingly, the relative autonomy index of the LSRQ was significantly higher in the innovation group at t_1_ (*M*_C_ = 2.26 ± 1.12; *M*_I_ = 2.66 ± 1.17; *p* = .037), with a standard error difference of 0.22 (Fig. 2D). The effect size was small (*d* = 0.35; 95% CI [-0.03, 0.74]).

Furthermore, we conducted a multiple linear regression analysis with controlled regulation (LSRQ) at the end of the course as the dependent variable. The independent variables considered were the study arm and the initial controlled regulation at t_0_ to control for preexisting individual differences (Supplementary material 1). The model demonstrated a high level of goodness-of-fit, with an *R*² of 0.44 (adjusted *R*² = 0.42). Both the initial controlled regulation at t_0_ (*p* < .001) and the study arm (*p* = .016) were significant predictors of the controlled regulation at t_1_, with the overall model being statistically significant (*F*(2, 102) = 39.36, *p* < .001). The β coefficients for t_0_ controlled regulation was 0.64, indicating that higher initial values were associated with higher controlled regulation at t_1_. The study arm had a β coefficient of -0.18, meaning that participants in the innovation group showed lower levels of controlled regulation at t_1_. The model’s statistical power was 1.00.

When conducting the same regression analysis for the relative autonomy index (LSRQ) at t_1_, only the initial relative autonomy index was a significant predictor (*p* < .001), while the study arm was only trend-significant (*p* = .064).

Among the variables assessed at t_1_, no statistically significant group differences were found regarding.

- IMI competence (*MD* = 0.08, *p* = .322).
- IMI pressure (*MD* = -0.18, *p* = .213).
- LSRQ autonomous regulation (*MD* = 0.05, *p* = .333).
- LCQ learning climate, with this questionnaire being trend-significant (*MD* = 0.21, *p* = .070).

When assessing freeform feedback in the post-course survey at t_1,_ we found a distinct difference regarding the participants’ perceptions of the group climate. Specifically, 19 of the students in the innovation group actively mentioned it as a positive aspect in their feedback, while only 7 in the control group did so. No negative remarks were made in either group.

### Lower stress levels in the innovation group at the end of the course

**Fig. 3:**
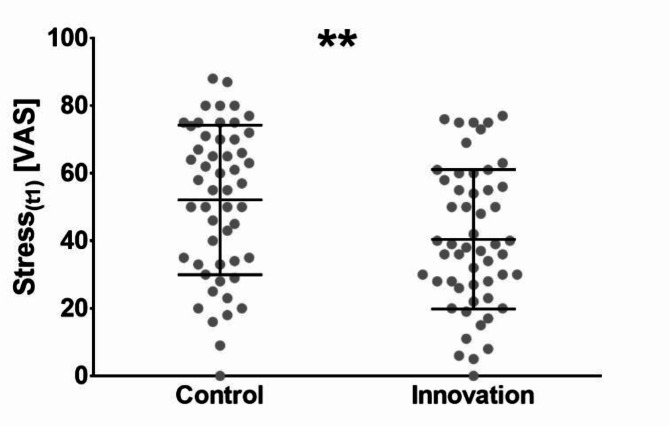
A significant difference between the control and innovation group was found regarding stress levels throughout the last week (*p* = .003) reported at t1 after the course on a visual analog scale from 0–100

Regarding our hypothesis that an SDT-based teaching concept could reduce students’ stress, a one-sided *t*-test revealed significantly lower stress levels in the innovation group after the course (*M*_C_ = 52.10 ± 22.15; *M*_I_ = 40.43 ± 20.65; *p* = .003), with a standard error difference of 4.18 (Fig. 3). The effect size indicated a medium effect (*d* = -0.55; 95% *CI* [-0.93, -0.15]).

To also account for stress levels reported at t_0_, which showed a trend-significant difference (*p* = .054) despite randomization, we conducted a multiple linear regression analysis considering the study arm and t_0_ stress as independent variables and t_1_ stress as the dependent variable (Supplementary material 2). The *R*² for the overall model was 0.27 (adjusted *R*² = 0.25), indicating a high goodness-of-fit. The regression analysis suggested that both stress levels at t_0_ (*p* < .001) and the study arm (*p* = .040) significantly contributed to predicting stress levels at t_1_ (*F*(2, 102) = 18.66, *p* < .001). The β coefficients for the independent variables were 0.45 for t_0_ stress and − 0.18 for the study arm, suggesting that higher stress before the course was associated with higher stress levels reported at the end, while being in the innovation group was linked to lower reported stress levels after the course week. A power analysis was conducted, and the statistical power of the model was estimated to be 0.99.

### Optimizing motivational factors reduced stress

We conducted further analyses to examine whether the favorable outcome regarding motivational factors facilitated the lower stress levels found among students in the innovation group.

**Fig. 4:**
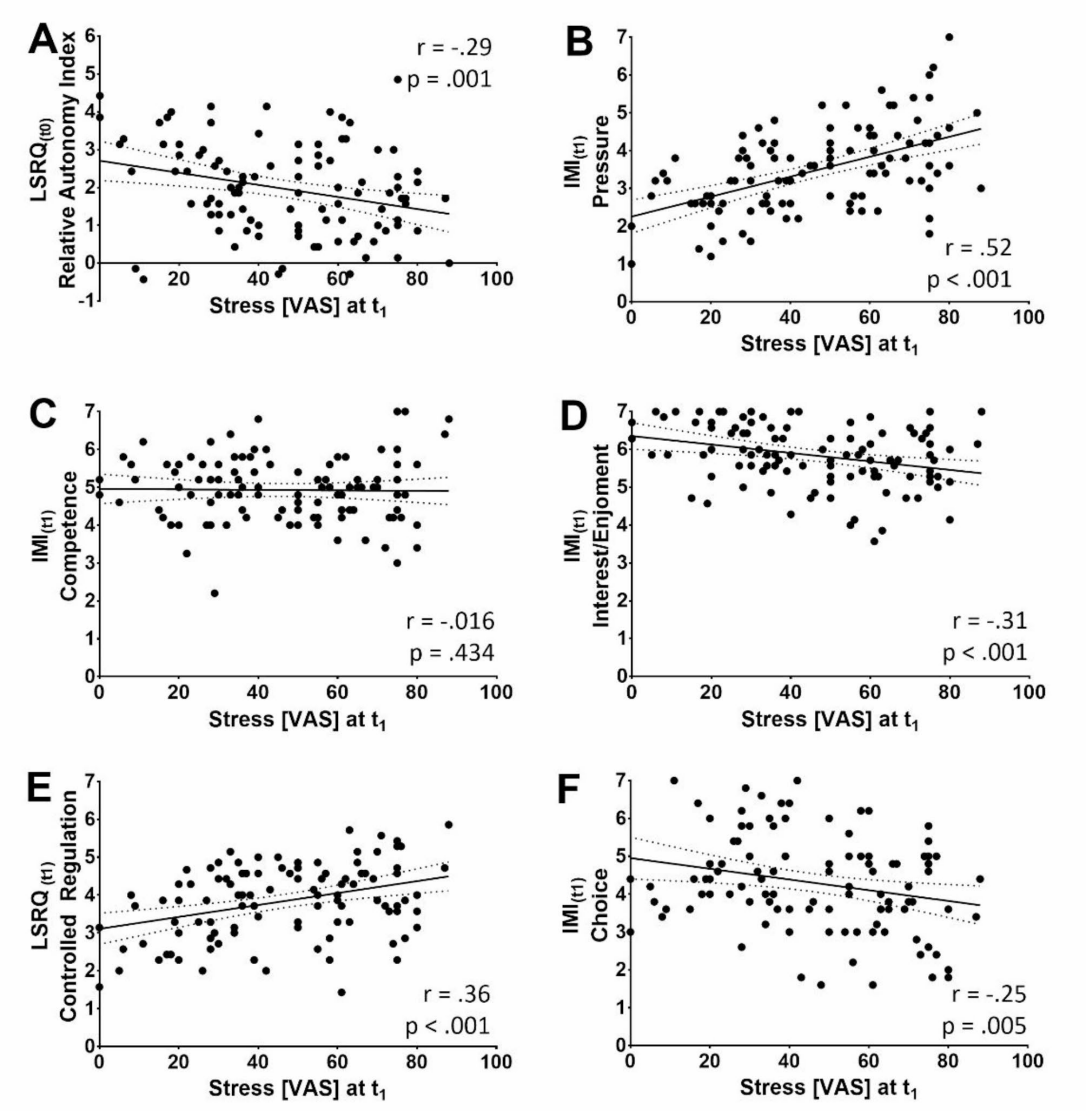
The learning self-regulation questionnaire (LSRQ) relative autonomy index at t0 before the course, which is calculated by subtracting the controlled regulation subscore from the questionnaire’s autonomous regulation subscore, showed a significant correlation with students’ stress reported at t1 after the course week. A significant correlation with stress at t1 was also found concerning the questionnaire’s controlled regulation subscale at t1. Furthermore, there were significant simple correlations between stress at t1 and the intrinsic motivation inventory (IMI) subscales of pressure, interest/enjoyment, and choice, whereas the competence subscale did not reach significance. Stress was measured on a visual analog scale from 0–100, while all other variables were reported on a 7-point Likert scale

To identify predictors of stress at t_1_ among the preexisting individual differences present at t_0_ and to examine the cumulative influence of the study arm yielded during the course, t_0_ stress, age, gender, t_0_ R-SPQ-2 F, t_0_ LSRQ, and the study arm were considered as potential independent variables in a multiple linear regression analysis (Supplementary material 3). Variables were included via a stepwise approach. The final model included 3 statistically significant variables, namely, t_0_ stress (*p* < .001), the t_0_ LSRQ relative autonomy index (*p* = .029), and the study arm (*p* = .040). With an *R*² of 0.30 (adjusted *R*² = 0.28), the model provided a strong model fit. The three variables were collectively significant in predicting t_1_ stress (*F*(3, 101) = 14.55, *p* < .001), with β coefficients of 0.41 for t_0_ stress, -0.19 for the t_0_ LSRQ relative autonomy index, and − 0.18 for the study arm. The model thus indicated that higher t_0_ stress predicted higher reported stress at the end of the course week (t_1_), while a more internalized locus of motivation (Fig. 4A) and participation in the innovation group were associated with lower stress reported at t_1_. The statistical power was 0.99.

To identify how factors assessed at the end of the course week were related to t_1_ stress, we constructed another model including t_0_ stress, age, gender, t_0_ R-SPQ-2 F (considered stable at t_1_), t_1_ LSRQ, t_1_ IMI, t_1_ LCQ, and the study arm as potential independent variables (Supplementary material 4). The study arm was included to account for the cumulative effect of our intervention otherwise not measured by the questionnaires collected at t_1_. Variables were again included using a stepwise approach (entry and removal criteria: *p* = .050).

IMI pressure (*p* < .001), t_0_ stress (*p* < .001), the study arm (*p* = .024), and IMI competence (*p* = .027) were eventually shown to be significant predictors of t_1_ stress. The model demonstrated a high level of goodness-of-fit with an *R*² of 0.67 (adjusted *R*² = 0.45). Collectively, the 4 variables were able to predict t_1_ stress with statistical significance (*F*(4, 100) = 20.42, *p* < .001). The respective β coefficients for these predictors were 0.46 for IMI pressure, 0.33 for t_0_ stress, -0.17 for the study arm, and 0.18 for IMI competence, suggesting that higher levels of perceived pressure (Fig. 4C) and competence (Fig. 4B) as well as higher stress at t_0_ predicted more elevated stress levels reported at t_1_, while being in the innovation group was associated with lower stress at t_1_. The statistical power was 1.00.

When excluding the study arm as an independent variable from this regression analysis under the assumption that it would have influenced the other variables at t_1_, the predictors included as significant remained the same, with the additional inclusion of t_1_ IMI interest (Supplementary material 5). This model demonstrated a high level of goodness-of-fit with an *R*² of 0.45 (adjusted *R*² = 0.43), and the 4 variables were able to predict t_1_ stress with statistical significance (*F*(4, 100) = 20.37, *p* < .001). The respective β coefficients for these predictors were 0.44 for IMI pressure, 0.35 for t_0_ stress, 0.22 for IMI competence, and − 0.19 for IMI interest, suggesting that in addition to our earlier findings, higher intrinsic motivation (Fig. 4D) was associated with lower stress at t_1_, indicating that this factor might have had the most influential effect on stress among the variables influenced by the innovation. The statistical power was 1.00.

We conducted a series of mediation analyses to explore whether the relationship between the study arm and students’ stress levels at the end of the course (t1) could be explained through differences in motivation.

**Fig. 5:**
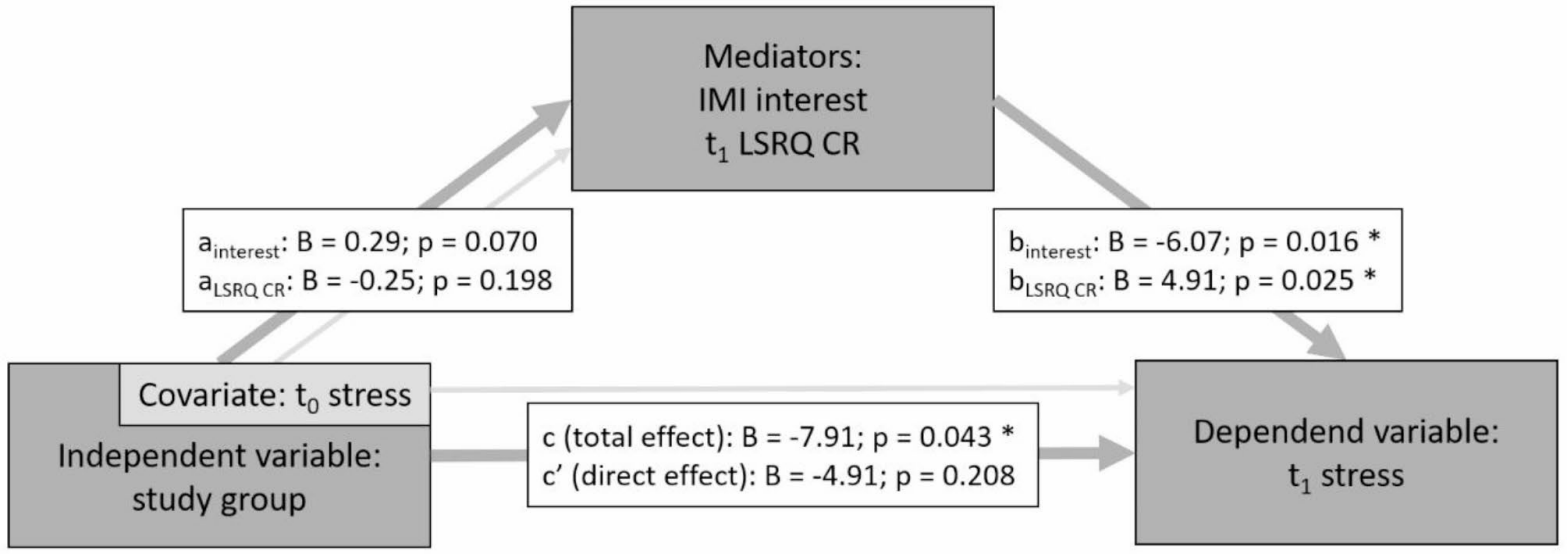
The relationship between the study arm and stress levels throughout the last week reported at t1 after the course week are fully mediated by intrinsic motivation measured on the interest/enjoyment subscale of the intrinsic motivation inventory (IMI interest) and the learning self-regulation questionnaire’s controlled regulation subscale (LSRQ CR) as parallel mediators. Both parallel mediators are measured on a 7-point Likert scale, and stress was reported on a visual analog scale from 0–100. Students’ stress reported at t0 before the course was taken into consideration as a covariate

An initial total effect of the study arm on t_1_ stress was observed (*B* = -11.66; *p* = .007). When t_0_ stress was added as a covariate to this model to account for the students’ baseline of stress before the course, the total effect of the study arm on t_1_ stress remained significant (*B* = -7.91; *p* = .043). The covariate t_0_ stress was also included in all further mediation analyses as a baseline control (Fig. 5).

The variables that our prior analyses had shown to be influenced by the study arm did not show significant mediation effects individually. However, the relationship between these variables and t_1_ stress was significant for IMI interest (*B* = -5.90; *p* = .019), t_1_ LSRQ controlled regulation (*B* = 4.754; *p* = .034), and t_1_ LSRQ relative autonomy index (*B* = -5.00; *p* = .009), while this was not the case for IMI choice.

Additionally, we tested models with multiple (parallel) mediators to investigate potential combined mediation effects.

When considering both IMI interest and t_1_ LSRQ controlled regulation simultaneously as mediator, the influence of the study arm on these two mediators, previously shown to be significant through *t*-tests and regression analyses, did not reach significance in this model. However, the impact of both IMI interest (*B* = -6.07; *p* = .016) and t_1_ SRC controlled regulation (*B* = 4.91; *p* = .025) on t_1_ stress was significant (Fig. 5).

There was no statistically significant direct effect of the study arm on t_1_ stress when accounting for the combined effect of interest and controlled regulation (B = -4.91; *p* = .208), indicating that the effect of the study arm on stress at t_1_ was fully mediated by these two variables. Furthermore, the total indirect effect, representing the overall mediation through IMI interest and t_1_ SRC controlled regulation, was significant and estimated at -2.99 (95%-*CI* [-6.56, -0.18]), which further supports a significant mediation. This finding suggested that higher levels of intrinsic motivation (Fig. 4D) and lower levels of controlled regulation (Fig. 4E) collectively mediated the relationship between students participating in the innovation group and them showing lower stress levels at t_1_.

When IMI choice (Fig. 4F) was added as a third mediator to the previous model, the total indirect effect of the three mediators was also significant, while the direct effect of the study arm on t_1_ stress was not, suggesting a full mediation. However, neither the effect of the study arm on IMI choice, nor the effect of IMI choice on t_1_ stress were significant in this combined model.

When replacing controlled regulation as a mediator with the relative autonomy index in either of the two mediation models, no significant indirect effects were found.

### Inferior OSCE results in the motivation group due to structural rather than individual factors such as stress or motivation

**Fig. 6:**
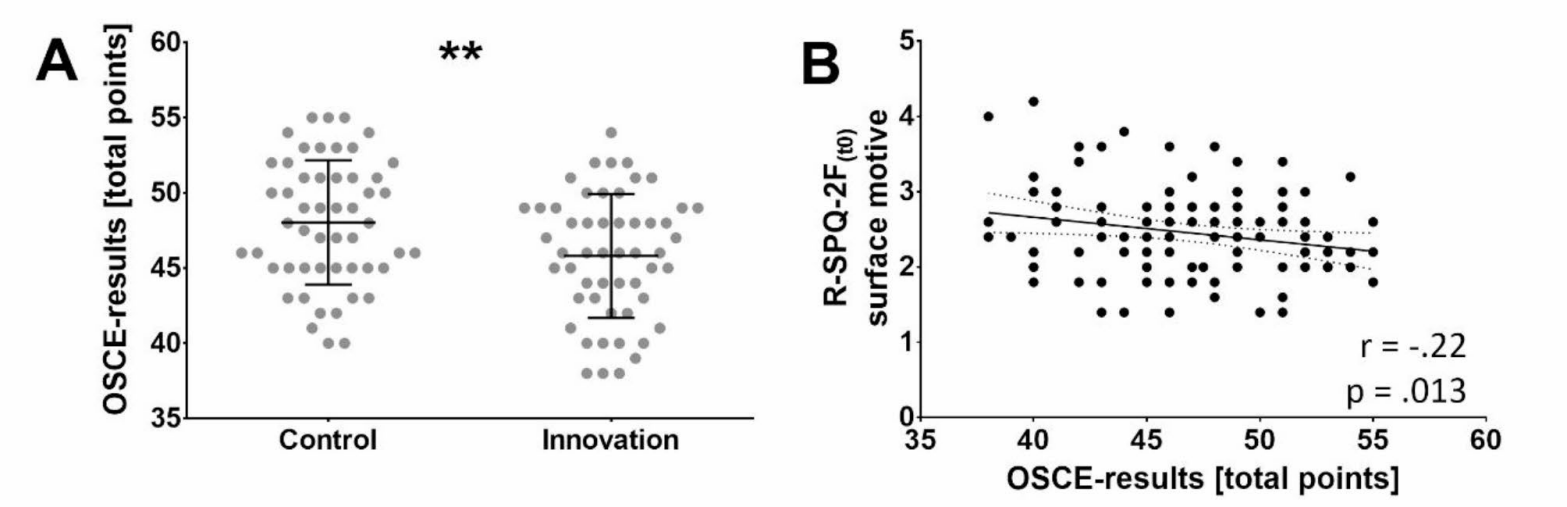
A significant difference between the control and innovation group was found regarding the exam score (*p* = .007) in the objective structured clinical exam (OSCE), which was graded from 0–60 at t2 on Monday after the course week. The revised 2-factor study process questionnaire (R-SPQ-2 F) subscale of surface motive, which is evaluated on a 5-point Likert scale at t0 before the course, shows a significant simple correlation with students’ exam scores in the objective structured clinical exam (OSCE), which was graded from 0–60 at t2 on Monday after the course week

When examining whether both study arms had achieved equal exam results, a two-sided *t*-test revealed significantly lower overall OSCE scores in the innovation group (*M*_C_ = 48.03 ± 4.14; *M*_I_ = 45.81 ± 4.12; *p =* .007), with a standard error difference of 0.81 (Fig. 6A). Notably, the significant difference also persisted when the most and least highly scoring topics and/or examiners were excluded as possible confounders. The effect size was medium (*d* = -0.54; 95% *CI* [-0.93, -0.15]).

To analyze the influence of preexisting individual differences present at t_0_ as well as the cumulative influence of the study arm on the OSCE exam results, we conducted a multiple regression analysis (Supplementary material 6). The study arm, gender, age, t_0_ LSRQ, t_0_ R-SPQ-2 F, and t_0_ stress were considered as potential independent factors. The OSCE result served as the dependent variable. We employed a stepwise approach for variable inclusion. The final model included the study arm and R-SPQ-2 F surface motive as independent variables and demonstrated a moderate goodness-of-fit, with an *R*² of 0.13 (adjusted *R*² = 0.12). Both the study arm (*p* = .002) and t_0_ R-SPQ-2 F surface motive (*p* = .007) as well as the overall model showed statistically significant predictive power for the OSCE result (*F*(2, 102) = 7.79, *p* < .001), with corresponding β coefficients of -0.29 for the study arm and − 0.26 for the R-SPQ-2 F surface motive. This finding suggested that being in the innovation group and having more surface learning motivation (Fig. 6B) were associated with poorer exam results. The statistical power was 0.95.

Simple and parallel mediations were performed to analyze whether this direct path of the study arm predicting students’ OSCE results was mediated by any of the group differences in motivation or stress that we observed. t_1_ IMI interest, t_1_ IMI choice, t_1_ LSRQ controlled regulation, t_1_ LSRQ relative autonomy index, and t_1_ stress, being the factors influenced by the study arm, were considered as potential simple mediators of the relationship between the study arm and the OSCE result (*B* = -2.22; *p* = .008). For parallel mediations, we considered both IMI interest and IMI choice in combination with either of the LSRQ variables as well as the respective models with t_1_ stress as an additional mediator. No significant total indirect effect of the mediator(s) was found in any of the models, and the direct effect of the study arm on the OSCE results remained significant in all models after adding the mediator(s), indicating that the relationship was neither partially nor fully mediated by stress or motivation.

Hierarchical linear modeling was employed to examine the predictive factors of students’ OSCE scores, with students (level 1) nested within teaching groups (level 2).

The intercept-only model with OSCE results as the outcome variable indicated that variance existed at both levels of the data structure (χ² = 60.38, *p* < .001). We found an intraclass correlation coefficient (*ICC*) of 0.71; thus, 71% of the variance in total OSCE scores was between groups, and 29% was between students within a given group.

We tested a combined level 1 and 2 model to examine which structural and individual factors had significant predictive value for the OSCE results.

Based on our prior results and hypotheses, we included the t_0_ R-SPQ-2F surface motive and surface strategy reflecting a surface learning approach, Δ stress, Δ LSRQ controlled regulation, and Δ LSRQ autonomous regulation to account for students’ baseline as well as final levels of stress and motivation and the exam topic as individual variables at level 1. At level 2, the study arm, time slot, lecturer, and examiner were included as structural variables. When testing this two-level model, the regression coefficients with robust standard errors relating the predictors to the OSCE results were significant for the study arm (*b* = -2.89, *p* < .001), time slot (*b* = -0.84, *p* = .002) and lecturer (*b* = 0.38, *p* = .016) at level 2 and the exam topic at level 1 (*b* = -1.03, *p* = .006). All other level 1 variables as well as the examiner (*b* = -0.08, *p* = .327) were not significant. This indicates that the exam results were effectively predicted by structural rather than individual factors and the exam topic. A decrease or increase in students’ stress or motivation as well as the degree of surface learning approach, on the other hand, had no significant predictive value. The predictors included in the combined HLM accounted for approximately 52% of the reduction in unexplained variance in the exam results (pseudo-*R*^2^ = 0.52).

By analyzing students’ free-form feedback, we found that 28 of the students in the innovation group (corresponding to 53%) mentioned that the exclusive use of strengths-based positive feedback hindered their learning process, while three students noted that it helped them feel more at ease. In the control group, six students mentioned that they enjoyed feedback, including positive and negative constructive criticism, while none mentioned that they were dissatisfied with the principle.

## Discussion

The data presented in this study show that a course concept optimized according to the SDT can increase intrinsic motivation for the task, foster students’ perceived autonomy, reduce controlled regulation, and ultimately mitigate the sense of stress in medical students during a one-week psychiatric course. Contrary to our hypothesis, the course concept led to a weaker performance in the exam, which did however not seem to be a result of the differences in motivation and stress.

The innovation succeeded in promoting an improved sense of perceived choice, as a measure of autonomy, being one of the three targeted base factors strengthening intrinsic motivation according to the SDT.

The IMI revealed superior intrinsic motivation among medical students taught according to the new concept, which was shown to play a decisive role in reducing their stress levels. This reflects the importance of engaging students in an autonomy-supportive learning process by giving them task choice and providing task relevance and rationale to promote interest in a subject and vice versa [42], including effects as far-reaching as the choice of their future specialty [14, 43, 44].

We also found a shift in the balance from autonomous to controlled regulation, while improvements in autonomous regulation itself did not reach significance in the LSRQ. The fact that the IMI detected significantly higher intrinsic motivation in our innovation group, while the LSRQ did not show a significant difference for autonomous regulation between the study arms, reflects the intricacy of measuring changes in intrinsic motivation over a short period. In our study, this might additionally be explained by the already high scores on the LSRQ subscale for autonomous regulation in both groups at t_0_, possibly leading to a ceiling effect.

We did not observe significant improvements in perceived competence or relatedness, as the other two targeted SDT factors, or in pressure. Regarding relatedness, students’ freeform feedback did suggest that the innovation group experienced an improved group climate. With the LCQ focusing mainly on relatedness to and autonomy support by the lecturer, our questionnaires might not have been able to detect this difference. The IMI subscale of “relatedness”, which has not been fully validated according to the Center for Self-Determination Theory, has shown satisfactory validity in some studies [45, 46] and might be able to better assess the targeted changes in future studies.

The innovation’s impact on stress perception was notable, with significantly lower stress reported in the innovation group after the course than in the control group. This was also true when accounting for differences in pre-course stress levels. Successful initiatives to reduce stress in an educational setting at medical schools or universities in general have been described by various studies, with the most effective methods being mindfulness practices, cognitive-behavioral and relaxation strategies, and social ability training [47–49]. The positive correlation between intrinsic motivation and mental well-being (including lower stress levels) is well established, and it is known that they can successfully be promoted bidirectionally by the aforementioned measures [50]. Nevertheless, research examining active approaches to promoting motivation with the explicit aim of thereby reducing stress in university students is limited.

Prior research indicates that stress levels in university students are influenced by a range of internal and external factors [51], among which cognitive processes and self-esteem [52] as well as the SDT and the job demands-resources model [53] have been described as key factors in understanding and counteracting students’ distress. As anticipated, we found that heightened intrinsic motivation mitigated stress, whereas increased controlled regulation exacerbated stress levels. This seemingly mediated the positive effect of the didactical innovation on stress levels reported at the end of the course. This finding aligns with previous research showing that greater autonomy support promotes positive affect and better performance through autonomous motivation, while increased self-criticism leading to negative affect is mediated by controlled motivation [54]. Although we identified perceived competence and pressure to be among the most impactful predictors of stress at t_1_, our innovation did not succeed in significantly improving these factors by providing only strengths-based positive feedback. Therefore, other methods should be explored to that end. Furthermore, greater perceived competence was associated with heightened stress, seemingly contrary to SDT, possibly reflecting greater self-imposed expectations to perform well.

Our parallel mediation analysis indicated that intrinsic motivation and controlled regulation fully mediated the relationship between the study arm and stress levels at t_1_. However, the lack of significance of the relationship between the study arm and the mediators in this combined model suggested that the changes induced by our innovation might not have been substantial enough to unequivocally determine their effects and interactions. While the innovation aimed to cater to students’ basic psychological needs, it is possible that not all SDT domains were addressed with enough depth or specificity to produce significant effects. For instance, autonomy-supportive teaching involves a broad range of actions, from offering meaningful choices to encouraging initiative and self-regulation, which may not have been fully implemented across all dimensions of the intervention [5, 55]. It is furthermore plausible that the interactions between a learning environment, motivational factors, and stress extend beyond the effects directly measured in our study and that other influential factors may have contributed to our results. Other studies have likewise found that mediating effects concerning intrinsic motivation and the satisfaction of basic psychological needs are multifaceted [56–59]. Longitudinal studies with multiple assessment points over extended periods as well as in diverse settings could provide deeper insights into the complex interactions between autonomy-supportive teaching, the satisfaction of students’ basic psychological needs, and their motivation, stress, and performance.

Contrary to our hypothesis that our new course concept would not affect students’ performance in the OSCE, the innovation group achieved lower exam scores. However, our mediation and HLM results suggested that this was likely due to structural aspects, such as the lecturer, exam topic, time slot within the semester and the course concept itself. Neither the lower controlled regulation nor the lower stress levels in the innovation group appear to have led to a decrease in students’ performance. In line with SDT, this reflects that a focus on external rewards does not foster better academic performance, which corresponds to earlier research showing that successful performance in an exam is not promoted by external regulation [9]. Our finding that a greater degree of the R-SPQ-2 F’s surface learning motive was a significant negative predictor of exam results further suggests that having extrinsically motivated reasons for learning a subject does not lead to better academic performance. In fact, students with stronger surface motives performed worse than their peers who had fewer surface motives. A possible explanation for the weaker exam performance was brought up in the freeform feedback contributed by the students themselves: a majority of the students highlighted the lack of critical feedback as hindering their ability to address their weaknesses precisely and to work toward improving their interviewing skills more effectively. Generally, receiving feedback is a useful tool for improving performance [60–63]. While exclusively positive feedback has been found to be beneficial to both performance and emotional responses in a workplace environment [64], the feedback we received rather reflects the theory that in particular high-achieving students benefit from feedback that challenges them [65]. The innovation group may have stayed within a “comfort zone” due to a lesser degree of challenging guidance to help them make difficult but achievable progress limiting in a more demanding “learning zone”, in line with the zone of proximal development theory [66]. Furthermore, the exclusively strengths-based positive feedback might have made students overconfident in their skills, which in turn might have led to them placing less focus on improving and preparing for the exam. While unbalanced negative critique can deter students’ motivation [29], criticism can also be motivational, when given with suggestions for future improvement and encouragement regarding the student’s ability to improve [67]. Therefore, the preexisting feedback method used in our control group, which included both positive reinforcement and constructive criticism, was likely already aligned with SDT principles. Our new approach may only constitute an improvement in settings where feedback is imbalanced or overly critical. In general, the effectiveness of feedback is highly context- and method-dependent [29, 60, 62, 63] Its influence on cognitive outcomes seems to be stronger than that on motivational and behavioral outcomes [62], possibly making other factors in educational concepts more suitable targets for enhancing motivational aspects and reducing pressure. Another interpretation of our findings could be that each student conducting only one to two interviews might not have given them enough time and opportunity to understand their mistakes, weaknesses, and potential for improvement through self-reflection and abstraction via model learning when exclusively giving strengths-based positive feedback. This finding corresponds to observations that positive-only feedback is more effective when applied to longer tasks rather than to shorter ones [68]. Complementing strengths-based positive feedback with constructive, change-oriented feedback delivered in an autonomy-supportive manner might function as the most effective approach because it encourages and motivates students while enabling them to make objective progress and simultaneously gain a sense of accomplishment. This would align with earlier findings with a focus on medical education [61, 69] as well as general higher education [70]. Moreover, research suggests that the way feedback is delivered can significantly impact student motivation and that autonomy-supportive feedback, which provides a clear rationale, acknowledges the student’s perspective, and fosters both competence and autonomy, is key to enhancing intrinsic motivation [71, 72].

Despite our students’ feedback and our data aligning with the theory that using exclusively strengths-based positive feedback did not lead to the desired outcomes, we cannot conclusively rule out that it might still have had a positive influence on students’ motivation and stress. To determine each method’s individual effects, further research with multiple study arms and partial implementation of the didactic innovation would be needed.

Further limitations in our study include the potential bias introduced by self-reported measures, retesting questionnaires, and the study’s singular focus on a psychiatric clinical setting. We found an even distribution of population characteristics in the two groups; however, despite randomizing and evenly distributing course groups to account for exam periods with generally greater stress, there may be additional decisive factors that we did not register. The effects observed in our study, given its relatively short duration, may not fully represent the long-term outcomes expected in longer longitudinal studies. Further research would be needed for validating our results over extended periods. Furthermore, the impact of motivation on questionnaire response rates could lead to potential biases through data collection, specifically when examining motivation as an outcome variable in the study; however, the return rates of the control group and innovation group were nearly equal.

Potential inconsistencies with the implementation of our teaching methods by different lecturers were counteracted by briefing them on the procedure of the respective group before the start of each week, accompanied by a handbook with precise instructions, by the randomization of the lecturers’ assignment to teaching groups and by matching those that taught more than one teaching group to both study arms.

## Conclusions

In summary, our didactical concept, which was designed in accordance with SDT to promote the basic psychological needs of autonomy, competence, and relatedness, positively impacted motivation and stress in line with our expectations. The weaker OSCE performance of the innovation group seemed to be rooted in a lack of critical feedback, although further research is needed to explore to what extent that finding is valid in other educational settings. Our data, consistent with previous research, suggest that pairing positive strengths-based positive feedback with constructive criticism might be most efficient in providing the conditions for optimal academic performance.

The complexity of the relationships between motivation, stress, and performance highlights the need for a deeper understanding of their multifaceted nature and how they can be positively influenced within medical as well as general education. Incorporating multidimensional assessment tools and conducting longitudinal studies will be necessary to comprehensively examine their relationships and interactions. In light of this complexity, the results of our study call for future research to investigate the transferability of innovative SDT-optimized teaching methods to other medical specialties as well as to non-medical education. Meanwhile, they give us grounds to be optimistic that teaching concepts aligned with SDT can be an important step toward the promotion of intrinsic motivation and stress reduction. Based on our findings, educators can effectively improve learning conditions by integrating SDT-based didactic methods into already existing curricula. For instance, simply offering students more task choices and opportunities for self-regulation or creating a learning space where they can feel part of a group could help foster intrinsic motivation while reducing stress. To best support students’ motivation and performance, educators can pair strengths-based feedback with critical, constructive criticism. Eventually, focusing on providing a learning experience that meets students’ basic psychological needs and fosters motivation can help them thrive in their educational environment.

## Electronic Supplementary Material

Below is the link to the electronic supplementary material.

Supplementary Material 1

## Data Availability

The datasets used and/or analyzed during the current study are available from the corresponding author upon reasonable request.

## Abbreviations

SDT: Self-determination theory
HLM: Hierarchic linear modeling
RCT: Randomized controlled trial
OSCE: Objective structured clinical exam
LSRQ: Learning self-regulation questionnaire
R-SPQ-2F: Revised 2-factor study process questionnaire
IMI: Intrinsic motivation inventory
LCQ: Perceived autonomy support learning climate questionnaire
VAS: Visual analog scale
