# Supplementary material for: Promoting motivation and reducing stress in medical students by utilizing self-determination theory – a randomized controlled trial in practical psychiatry courses
Source: BMC Med Educ. 2024 Oct 19;24:1177. doi: 10.1186/s12909-024-06181-9 (PMC11491017; doi:10.1186/s12909-024-06181-9)
Supplement: Supplementary file 1 — Supplementary Material 1 [file 12909_2024_6181_MOESM1_ESM.docx]

**Supplementary Materials**

Supplementary material 1:${SRQcontrolled\_regulation}_{t1} = 0.64 * {SRQcontrolled\_regulation}_{t0} - 0.35 * study arm + 1.46$

Supplementary material 2:${stress}_{t1} = 0.40 * {stress}_{t0} - 7.91 * study arm + 31.12$

Supplementary material 3:${stress}_{t1} = 0.36 * {stress}_{t0} - 3.47 * {SRQrelative\_autonomy\_index}_{t0} - 7.74 * study arm + 39.63$

Supplementary material 4:${stress}_{t1} = 9.28 * IMIpressure + 0.30 * {stress}_{t0} - 7.62 * study arm + 4.56 * IMIcompetence - 18.65$

Supplementary material 5:${stress}_{t1} = 8.52 * IMIpressure + 0.31 * {stress}_{t0} + 5.72 * IMIcompetence - 5.09 * IMIinterest + 3.31$

Supplementary material 6:$OSCE = -2.49 * study arm - 1.84 * SPQsuperficial\_motive + 52.70$
